# Automated vocabulary discovery for geo-parsing online epidemic intelligence

**DOI:** 10.1186/1471-2105-10-385

**Published:** 2009-11-24

**Authors:** Mikaela Keller, Clark C Freifeld, John S Brownstein

**Affiliations:** 1 Children's Hospital Informatics Program at the Harvard-MIT Division of Health Sciences and Technology, 300 Longwood Ave, Boston, MA 02115, USA; 2 Department of Pediatrics, Harvard Medical School, Boston, MA 02115, USA

## Abstract

**Background:**

Automated surveillance of the Internet provides a timely and sensitive method for alerting on global emerging infectious disease threats. HealthMap is part of a new generation of online systems designed to monitor and visualize, on a real-time basis, disease outbreak alerts as reported by online news media and public health sources. HealthMap is of specific interest for national and international public health organizations and international travelers. A particular task that makes such a surveillance useful is the automated discovery of the geographic references contained in the retrieved outbreak alerts. This task is sometimes referred to as "geo-parsing". A typical approach to geo-parsing would demand an expensive training corpus of alerts manually tagged by a human.

**Results:**

Given that human readers perform this kind of task by using both their lexical and contextual knowledge, we developed an approach which relies on a relatively small expert-built gazetteer, thus limiting the need of human input, but focuses on learning the context in which geographic references appear. We show in a set of experiments, that this approach exhibits a substantial capacity to discover geographic locations outside of its initial lexicon.

**Conclusion:**

The results of this analysis provide a framework for future automated global surveillance efforts that reduce manual input and improve timeliness of reporting.

## Background

Web-based information sources such as online news media, government websites, mailing lists, blogs and chatrooms provide valuable epidemic intelligence by disseminating current, highly local information about outbreaks, especially in geographic areas that have limited public health infrastructure. These data have been credited with providing early evidence of disease event, such as SARS (Severe Acute Respiratory Syndrome) and avian influenza [1]. The availability of open source and freely available technology has spawned a new generation of disease "mashups" that scour the web and provide real-time outbreak information. HealthMap [2,3] is one such system that monitors, analyzes and disseminates disease outbreak alerts in news media from all around the world. Each hour, the system automatically queries over 20,000 sources using news aggregators such as Google News, for relevant reports. It filters the retrieved documents into several taxonomies and provides on its website, http://www.HealthMap.org, a geographic and disease-based display of the ongoing alerts. HealthMap provides a starting point for real-time intelligence on a broad range of emerging infectious diseases, and is designed for a diverse set of users, including public health officials and international travelers [4,5].

This real-time surveillance platform is composed of a number of Information Retrieval and Natural Language Processing modules, such as outbreak alert retrieval and categorization, information extraction, etc. In the present work we are interested in a critical task of the last phase of the information processing scheme: the geographic parsing ("geo-parsing") [6] of a disease outbreak alert or the extraction from one such textual document of its related geographic information. This information is needed for the precise geographic mapping, as well as for the identification/characterization of the particular disease outbreak described in the alert. Indeed each alert is uniquely characterized by its disease category, a set period in time and its precise geographic location. A good characterization of the outbreak allows the system to discriminate between duplications of an alert and new alerts. It is also essential for the evaluation of the system and for long-term analysis. Most importantly, the automated high resolution geographic assignment of alerts aids rapid triaging of important information by system users, including public agencies such as the World Health Organization and the US Centers for Disease Control and Prevention that use these data as part of their daily surveillance efforts.

So far, HealthMap assigns incoming alerts to a low resolution geographic description such as its country, and in some cases its immediately lower geographic designation (for the USA and Canada, it would provide for example the state or province). The system uses a rule-based approach relying on a specially crafted gazetteer, which was built incrementally by adding relevant geographic phrases extracted from the specific kind of news report intended for mapping. The approach consists in a look-up tree algorithm which tries to find a match between the sequences of words in the alert and the sequences of words in the entries of the gazetteer. It also implements a set of rules which use the position of the phrase in the alert to decide whether or not the phrase is related to the reported disease.

The gazetteer contains around 4,000 key phrases, some of which refers to geographic locations with several resolution levels (from hospitals' to countries'), some are negative phrases (≈ 500 phrases, *eg Brazil nut *or *turkey flock *are not considered location references) as well as phrases that are specific to the kind of data processed (*Center for Disease Control*, *Swedish health officials*, etc.).

While the current limited gazetteer has proven useful for a high level view of ongoing threats, there is a public health need to develop a method that provides the highest resolution geographic assignments, especially for public health practitioners that require this information for outbreak verification and follow-up. The approach presented in the following section is an attempt at producing a geo-parser using the prior knowledge encoded in the gazetteer as a base. At first glance, it would seem that in order to increase the resolution of the HealthMap geo-parser, expanding the size of the gazetteer should be enough. However in our experience, adding new terms to the gazetteer, without careful supervision often results in an upsurge of false positives for the system. The ability to predict statistically if a word in a sentence is a geographic reference is a valuable feature for a geo-parser. Indeed no matter the size of the gazetteer, it cannot contain every geographic reference, and even words in the gazetteer might be false positives in the alert (*Canada geese*). The typical way of obtaining a statistical predictor is to have access to annotated training data. However, data annotation is a very time consuming and expensive task, and thus the approach we present is an attempt at circumventing it by using the already available gazetteer.

The inspiration behind the present approach is based on the intuition that a human reader presented with a text containing a phrase that is out of his vocabulary would most likely be able to guess whether this phrase refers to a geographic location or not. This reader would infer the semantic role of the phrase with a certain accuracy, because he has prior knowledge of the syntactic context in which geographic references appear, maybe also of their particular character distribution or the fact that they generally begin with a capital letter, etc. Our approach in some sense simulates this situation with a learning algorithm in the guise of an artificial "reader." We use the HealthMap gazetteer as the supervision (or reference) for training the reader. Some of the location words in the training texts are purposely hidden from the reader's vocabulary in order to divert the attention of the learning algorithm to the context on which these words appears instead of the words themselves. Previous related natural language processing approaches reported in the literature, like ours, use a limited knowledge base to generate a broader one.

The task we are trying to solve, namely finding geographic location references in a text, falls into the more generic Natural Language Processing problem of Information Extraction [7-9], which involves automatically selecting sub-strings, containing specific types of information, from a text. It is in particular closely related to the Named Entity Recognition task [10], in which texts are parsed in search of references (mainly proper names) to persons, organizations and locations or more recently gene name [11]. However, we are here interested in more than just named entities, since any hint of location, even, for example, adjectives (*French authorities*) or public health organizations (*INSERM*: a French institute of medical research), can provide us with desired information.

There have been a number of approaches to named entity recognition and more generally to information extraction problems (see *eg *[12,13] or [14] for a name entity recognition system being used in biosurveillance), exploiting as we do, syntactic and contextual information. They however usually rely on supervised approaches, which require heavily annotated datasets to account for the human experience. Building these annotated corpora, is extremely time-consuming, expensive and results in a so-called knowledge-engineering bottleneck. On the other hand, large numbers of unlabeled texts are easily available through, for example, the Internet.

In order to take advantage of the unlabeled data, while avoiding the cumbersome need of annotation, a number of approaches, sometimes referred to as Automatic Knowledge Acquisition [15] have been developed. The domains to which each of these methods is applied are very diverse. Some concentrate specifically on the named entity classification problem [16,17], while others, like ours, have a different information extraction scope [15,18,19]. Whether they use a few rules [16,19] (*eg Mr. [Proper Noun] *→ *Person*) or a small lexicon [15-17,20] (such as a small gazetteer) as seeds for the information to be extracted, all approaches, including ours, begin with a corpus partially labeled. These approaches are related to semi-supervised learning, where a few labeled examples are used in conjunction of a large number of unlabeled ones. The goal of all these models is to exploit the redundancy of language and to learn a generalization of the context in which labels appear.

Strategies on how to use these few labeled examples diverge. Most of these approaches go through their training sets in several steps and incrementally add inferred labels to their labeled examples, in a bootstrap fashion, [15-18,20]. Of course, the addition of every inferred labeled example (even false positives) can quickly produce a drift towards a noisy solution. These approaches have thus heuristics to decide which examples to add at each iteration. Other approaches, like ours, are built to learn everything they need from the initial input only [19].

While our present work focuses on geo-parsing an English-language corpus, HealthMap surveillance so far covers alerts in 4 more languages (Spanish, French, Russian and Chinese), and plans to expand to other languages. Most of these approaches, [15,16,19,20], use elaborate linguistic knowledge either to represent the words or to target groups of words to tag. Relying on complex linguistic features requires language-specific expert knowledge difficult to obtain. Our approach relies on low level syntactic features making it easily portable to other languages. In addition, it is based on statistical machine learning principles (like [16,17]), as opposed to rule-based ones, which also reduces the need of expert knowledge.

## Results

### Core idea

**Figure 1:**
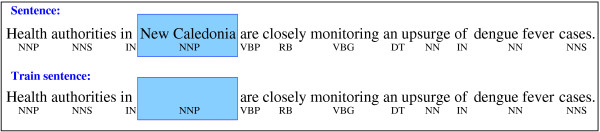
**Example of training data**. An illustration of the word-withholding strategy.

The core idea behind our approach is to have a dataset of alerts tagged with the gazetteer-based algorithm as well as with more general linguistic knowledge (*eg *part-of-speech tags, etc.), and then to use this dataset with tags partially hidden to learn a generalization of the parsing. In the example of Figure 1, a sentence is annotated with its corresponding part-of-speech tags and gazetteer-based geo-parsing tags (the blue rectangle). In order to learn a generalization of the geo-parsing scheme, the same sentence would be used in our training dataset with the specific identity of the word *New Caledonia *hidden, but its part-of-speech preserved.

### Additional features

Our dataset consists of English-language disease outbreak alerts retrieved in 2007 by the HealthMap system. We used the HealthMap rule-based approach to tag the words in the alert that match geographical references found in the gazetteer. To enrich the dataset with syntactic information, we tagged the alerts with a part-of-speech tagger (see Methods section for more details). Provided that, in English, location names often begin with capital letters or appear as acronyms, we assigned to the words in the alerts, in addition to their part-of-speech tags, a capitalization status, *ie *none, first character, upper case.

**Figure 2:**
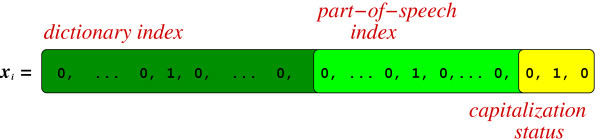
**Words sparse representation**. Each word in the dictionary, its part-of-speech in the text and its capitalization status are associated with 3 indexes in the representation. If a word is out of the dictionary, it will only be represented by its part-of-speech and capitalization status.

From a more formal perspective, our dataset (or corpus) is composed of *P *examples {(**x**_1_, **y**_1_), ..., (**x**_*P*_, **y**_*P*_)}. Each example (**x**, **y**) is composed of an alert **x **= [*x*_1_, ..., *x*_*L*_] with a certain number *L *of words *x*_*i*_, and their corresponding "location" labels **y **= [*y*_1_, ..., *y*_*L*_], *y*_*i *_∈ {*loc, none*}, that is, *y*_*i *_is (part of) a location reference or not. The words *x*_*i *_are represented by their part-of-speech tag, their capitalization status and occasionally by their index in a dictionary **D**, extracted from the training dataset. Figure 2 illustrates the vectorial representation of words. The |**D**| (size of **D**) first components of *x*_*i *_correspond to the dictionary indices and are all equal to zero, except for the position coinciding with the word index in **D**. Similarly, the next *K *features of *x*_*i *_correspond to the part-of-speech tag indices in the *K *part-of-speech tag list. And finally, the last three features stand for the three possible capitalization values.

### Hiding words

As explained previously, one important characteristic of this experiment is the fact that the words (that is, the lexical information) are only partially accessible to the learning algorithm. This is implemented by the choice of the dictionary **D **mentioned in the previous paragraph. As we will explain shortly, some words in the corpus are purposely left out of the dictionary. Consequently, the first components of their sparse representation, the ones referred to as dictionary index in Fig. 2, are all set to zero. Such words are thus represented only by their part-of-speech tag and their capitalization status.

**Figure 3:**
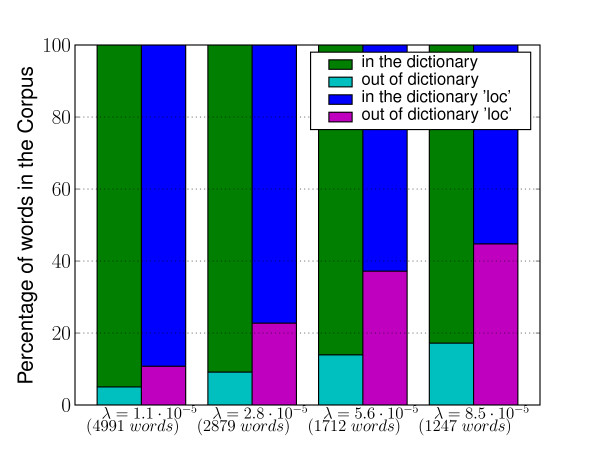
**Percentage of artificial out-of-vocabulary words**. Percentage of "hidden" words when reducing the dictionary size according to the minimum frequency thresholds *λ*. The first bar at each *λ *value shows the number of out-of-vocabulary words among the words of the corpus, and the second bar shows the number of location words outside the vocabulary among the words tagged as location references using the HealthMap gazetteer. Between brackets, the dictionary size corresponding to *λ *is reported.

**Figure 4:**
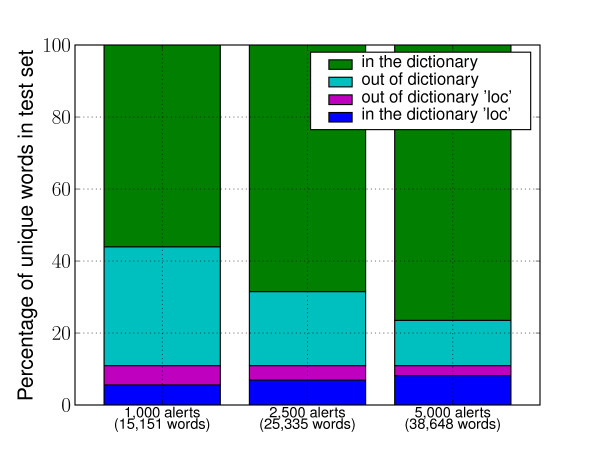
**Percentage of natural out-of-vocabulary words**. Percentage of unique words from a separated evaluation set (500 alerts, 11,184 unique words) that are inside or outside of the training set extracted dictionary, for training sets *T*_0 _(1,000 alerts), *T*_1 _(2,500 alerts) and *T*_2 _(5,000 alerts). The percentage of location words is computed with respect to the locations found by the commercial geo-parser (see sect.).

How we choose which words to hide is critical to the generalization power of our learning algorithm. Indeed, naively deciding to hide the words tagged as location by the HealthMap gazetteer would be equivalent to giving away the label information to the learning algorithm. It would lead it to learn "by heart" what it is that the gazetteer considers a location, while what we are interested in is making it discover new location patterns. The strategy we implemented is based on the idea that words referring to specific locations have a lower frequency (*ie *number of occurrences/corpus size) (average frequency = 4.9/10^5^(± 12.3/10^5^)) in the corpus than "typical" words (average frequency = 8.9/10^5^(± 103.3/10^5^)). For example, the word *authorities *appears 268 times in our 1,000 alerts corpus (≈ 355,000 words) while, the word *Australia *appears only 38 times. Continuing the metaphor of the artificial reader, we decide to cut words out of its vocabulary indiscriminately, based solely on their frequency. We make the reasonable assumption that if a word is rare then it is less likely to be "known" by the reader. Figure 3 shows that applying this strategy indeed leads to hiding more location words than "typical" words. As shown in the figure, for a given word's frequency cutoff, the percentage of hidden words out of all words in the corpus (first bar) is lower than the percentage of hidden location words out of all location words (second bar). The graph shows, for example, that cutting words that appear with a frequency lower than 2.8 × 1*e*^-5^, hides roughly 10% of the words in the corpus, but close to 25% of the location word occurrences (≈ 2-3% of the words in the corpus are location words). Lets us call this lower bound on the word frequency *λ*. We apply *λ *onto the corpus to decide whether or not a word index is to be hidden, so that only frequent word indices are visible to the model. Another justification of our approach is the following. The list of words present in the training set represents the largest vocabulary the algorithm has access to. In general, it would seem a good idea to diversify and augment this vocabulary, by increasing the size of the training set, so that the algorithm is presented with examples of usage of the new words - as is typically done in a fully supervised approach. Since we do not have access to fully annotated corpora, but rely on a finite gazetteer to label the data, increasing the size of the training set would eventually be redundant. From another point of view, no matter the size of the training set, there is no guarantee that all words in a new set of texts will fall within the algorithm's dictionary. As an example, Figure 4 shows the percentage of out-of-vocabulary words among the unique words of an evaluation set, for several vocabulary sizes. Our approach reduces the dictionary available to the learning algorithm, placing it, during the training, in the situation that it would eventually face when confronted with new data. In particular, since we reduce the dictionary in a way that respects the distribution of words, we make the hypothesis that the location words that we purposely keep out of the dictionary can work as surrogates of the new location words we want to discover.

Using the data just described, we trained an artificial neural network to output a probability estimate of the label value *y*_*i *_for the *i*^*th *^word *x*_*i *_in an alert **x**,

given a window (*n *- 1 = 2 × *hw*) of preceding and following words. A threshold on *NN*(*i*, **x**) allows us to decide if the input is a location or not. The neural network was trained by negative log-likelihood minimization using stochastic gradient descent. An extensive description of the learning algorithm architecture and optimization is provide in the "Methods" section.

### Evaluation

The same lack of labeled data that we were faced with for training the geo-parser applies to the question of how we can test the performance of a trained model. Indeed, to measure the accuracy of the outputs of the geo-parser, we would need "correct" annotations to compare with. Ideally, we would even have a test corpus annotated by several humans independently and thus be able to measure the difficulty of the task we are trying to solve. As stated previously, annotating such a dataset is a tedious and expensive task and we thus consider it as last resort. In order to nonetheless evaluate the performance of the algorithm we devised two approximate solutions.

• First, as in the training phase we can reuse the HealthMap gazetteer to tag the test corpus and then evaluate how much of the gazetteer annotation the neural network geo-parser (or NN geo-parser) is able to recover. It might seem that retrieving the locations that were used for training is an easy task. However, the same lexical information that was hidden in the training corpus would be hidden in the test dataset as well. As a consequence, both training and test examples provide only general context information, and thus rediscovering the HealthMap gazetteer labels is not such a trivial task.

• In a second experiment, we used a comprehensive (subscription only) commercial geo-parser (MetaCarta GeoTagger [21]) to tag 500 alerts with what we would consider "true" location references. Note however that despite the fact that there is good overlap between the HealthMap gazetteer and the MetaCarta tags, there are also a certain number of tags that are found only in the HealthMap annotation. Taking both sets of tags as a whole: 52.4% are found MetaCarta's only, both geo-parsers agree on 38.7% of the tags and the remaining 8.9% come from the HealthMap gazetteer. Some of the HealthMap only tags are due to minor uninteresting variations in the annotation schema, while others suggest a specialisation of the HealthMap gazetteer to public health content that the more generic MetaCarta geo-parser is obviously not trained to provide.

We trained several neural network geo-parsers on the three datasets *T*_0 _(1,000 alerts), *T*_1 _(2,500 alerts) and *T*_2 _(5,000 alerts), with extracted dictionaries of varying sizes according to our lower bound *λ*. Given the approximate nature of the solution found when training neural networks by stochastic gradient descent, we repeated the learning process for each condition 5 times to estimate the variance. The evaluation corpus contains 500 disease outbreak alerts subsequent to the ones used for training, in respect of the temporal nature of the HealthMap surveillance process. This represents 201,643 words to tag among which 5,030 are words that are considered locations by the HealthMap gazetteer approach, 7,385 are considered locations by the commercial geo-tagger, of which 3,315 by it alone (whereas 960 words are considered locations only by the HealthMap gazetteer approach).

**Figure 5:**
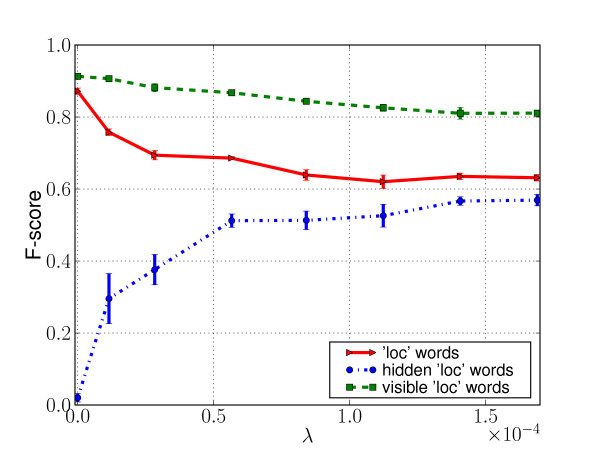
**Evaluation with respect to HealthMap gazetteer tags**. *F*_1 _score with respect to HealthMap gazetteer tags for several values of *λ *(red plain line). *F*_1 _scores among words with visible lexical index (green dashed line) and among words with hidden dictionary's index (blue pointed-dashed line)

Figure 5 displays the performance, with respect to the HealthMap gazetteer annotation, in terms of *F*_1 _score, of the NN geo-parser trained on *T*_1 _for increasing values of the lower bound *λ *on the word frequency (*ie *increasing numbers of words purposely kept out of the algorithm's dictionary). The *F*_1 _score, a measure of accuracy, is a combination of the precision,

(or, the proportion of correctly identified locations among the locations retrieved by the system, also referred to in certain fields as positive "predictive value of the system") and the recall of the system

(the proportion of retrieved locations among the "true" locations, or the "sensitivity" of the system):

To show that this approach is not just a memorization of the gazetteer-based approach, the results are sliced into the *F*_1 _scores of words inside and outside the algorithm's dictionary, *ie *words that were represented with and without their dictionary index feature. As the value of *λ *increases, the size of the dictionary we allow the NN geo-parser to see decreases and thus the number of words in the evaluation corpus that are out of the algorithm's dictionary increases. This makes the task of identifying which words in the text refer to locations more difficult, and as a visible consequence the overall performance of the system decreases (solid red line). On the other hand, however, the increase in out-of-dictionary examples greatly improves the ability of the system to correctly identify locations that are out of the algorithm's dictionary (dash-dotted blue line), ability that is non-existent when the whole training set vocabulary is available to the learning algorithm (*λ *= 0). As explained in previous sections, the idea behind this approach is to consider those purposely "hidden" location words as surrogates of the location words unknown to the gazetteer, those we want to be able to discover. The observed increase in performance in the retrieval of those words suggests that this approach would be appropriate for this task and without having too high a loss in performance for "visible" words (dashed green line).

**Figure 6:**
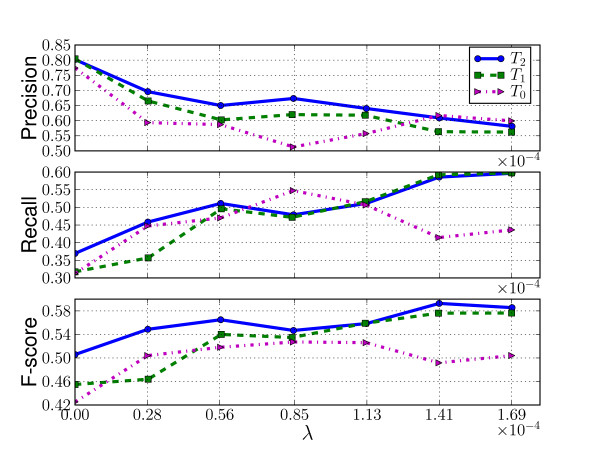
**Evaluation with respect to MetaCarta tags**. Precision, recall and *F*_1_-score with respect to MetaCarta labels for increasing dictionary cut-offs according to the *λ *threshold. Performances of models trained on *T*_0 _(1,000 alerts), *T*_1 _(2,500 alerts), *T*_2 _(5,000 alerts) and *T*_1 _with location and disease targets.

Figure 6 and Table 1 display results from the second experiment in which the performance of the NN trained on the training sets *T*_0_, *T*_1 _and *T*_2 _is compared to "true" location tags provided by the MetaCarta geo-tagger, on the same evaluation set as described previously. The performance of the NN geo-parsers are shown in Figure 6, while Table 1 lists the optimal *F*_1 _score obtained when the HealthMap gazetteer and the NN geo-parser are combined. The 3 graphs in Fig. 6 show that the word-withholding strategy has mainly a positive effect on the accuracy of the NN geo-parsers. While there is a decrease in precision, when the number of "hidden" words increases, the recall increases enough that their combination in the *F*_1 _score is higher than when the whole training vocabulary is used. Augmenting the size of the training set, as can also be seen in Table 1, also has a positive effect on the performance. However this positive effect seems to reach a plateau, since by doubling the number of alerts between *T*_1 _and *T*_2 _we only see a small improvement in *F*_1 _score.

**Table 1:** Best performances with respect to MetaCarta labels (optimal *F*_1_-score).

models	Prec	Rec	*F*_1_-score	*λ*.10^5^
HM Gaz.	**0.81**	0.42	0.56	-

Comb. *loc T*_0_	0.64	0.58	0.61 (± 0.006)	14.1

Comb. *loc T*_1_	0.63	0.64	0.63 (± 0.008)	11.2

Comb. *loc T*_2_	0.61	**0.68**	**0.64**(± 0.01)	14.1

It is worth noting that there are words that are here considered as false positives (and thus contribute to the decrease in precision) that are in fact generalizations of those words that HealthMap sees as location but that the more generic geo-parser ignores.

## Discussion

We have presented an approach to the geo-parsing of disease outbreak alerts in the absence of annotated data. The identification of precise geographic information in the context of disease outbreak surveillance from informal text sources (*eg *news stories) is essential to the characterization of the actual outbreaks, and would increase the resolution of a visualization scheme such as the one proposed by HealthMap. Such precise information extraction typically requires a dataset of texts with the desired information carefully annotated by a human expert. A corpus of this kind is expensive and time-consuming to create. Instead, we propose a statistical machine learning approach which generalizes the existing HealthMap rule-based geo-parsing by making use of the lexical and syntactic context in which the existing gazetteer phrases appear. We have demonstrated that the described model has indeed the ability to discover a substantial number of geographic references that are not present in the gazetteer. In addition, our approach also limits the number of false positive it produces.

Nevertheless, there is still a portion of those geographic references that remains a challenge to retrieve. There are several components of this approach that could be refined in order to improve the performance of the algorithm. For example, a more sophisticated word-withholding strategy could be implemented. In addition to relying on the term frequency, the strategy could also account for the part-of-speech of the word to hide (*eg *verbs are unlikely surrogates for locations), and on the alert frequency of the term (a word that appear a few times in the corpus but only in a unique alert might be more likely to be location than one with occurrences in several alerts). Another place for improvement could be the representation of words. Other features, aside from part-of-speech and capitalization, could provide additional information about the semantic status of the word. However, these features should be simple enough to implement in several languages, in order to comply with the requirement of portability formulated previously. Another further area of exploration, is the weight given to words tagged as *none *during training. Some of those words are actually unknown locations that, during the learning process, are strongly supervised as not being locations. Even if we compensate for this error by tuning the decision threshold, it should be also possible to act on the problem by a relaxation of the supervision during the training stage. Finally, the geo-parsing of an alert is only an intermediate step. Finding which terms in an alert are geographic references is crucial, but the final goal is to identify which among these terms is the definitive disease outbreak location and be able to disambiguate it (Paris, Texas vs Paris, France). Ideally, we would like to integrate these different tasks into one, so that the information that is learned from one can benefit the others.

From a more general point of view, the presented approach also describes a way of incorporating the prior knowledge encoded in a rule-based procedure into a more general statistical framework. This could be adapted to the extraction of other types of information that would also prove useful in the characterization of an outbreak. For example, we are interested in the extraction, from the alerts, of attributes related to the individuals involved in the outbreak such as age, sex, setting, clinical outcomes when specified.

## Conclusion

We have presented an approach to the geo-parsing of disease outbreak alerts in the absence of annotated data. The results of this analysis provide a framework for future automated global surveillance that reduce manual efforts and improve timeliness of reporting. Ultimately, the automated content analysis of news media and other nontraditional sources of surveillance data can facilitate early warning of emerging disease threats and improve timeliness of response and intervention.

## Methods

Our methodology can be summarized as follows. Using alerts retrieved by HealthMap we generated a dataset specially tailored to train a geo-parsing algorithm. To generate this dataset, the HealthMap gazetteer-based algorithm was first applied to this set of alerts in order to extract the words in the text referring to geographic locations. The same alerts were then run through the part-of-speech tagger algorithm provided by NEC's project SENNA (a Neural Network Architecture for Semantic Extraction) [22], making the syntax of the text explicit. This part-of-speech tagger has a reported accuracy of 96.85% on the reference Penn Treebank dataset [23]. We then assigned to every word in the alerts a capitalization status, *ie *none, first character, upper case. After these 3 steps in the data generation process, each word in each alert had 4 features: the word itself, its part-of-speech tag, its capitalization status and a label indicating if the word is a geographic location or not. The last step in the data generation process consisted in replacing the lexical feature of the words with lowest frequency by a blank, as explained in the Results section.

**Figure 7:**
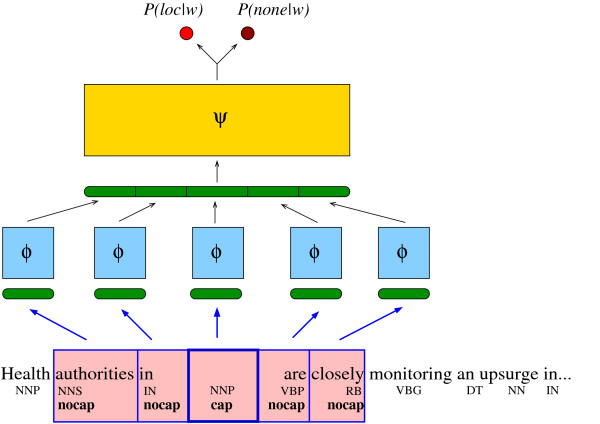
**Illustration of the neural network architecture**. An illustration of geo-parsing neural network with a typical input.

Using the data just described, we trained an artificial neural network to output a probability estimate of the label value *y*_*i *_for the *i*^*th *^word *x*_*i *_in an alert **x**,

given a window (*n *- 1 = 2 × *hw*) of preceding and following words. The neural network was trained by negative log-likelihood minimization using stochastic gradient descent. The neural network with an example of input (with *hw *= 2) is illustrated in Figure 7. The network architecture can be decomposed as follows. First, each word in the window sequence is given as input to a unique multi-layer perceptron (MLP) which has been replicated *n *= 2 × *hw *+ 1 times, in a siamese network fashion [24]. This first MLP can be seen as a function *ϕ *mapping the extremely sparse representation *x*_*i *_of the words into a new representation, *ϕ*(*x*_*i*_) ∈ **R**^*d*^, which has the advantage of being learned during the training. This approach was applied with success for language modeling in [25] and more recently for semantic role parsing in [22].

The outputs *ϕ*(*x*_*i*-*hw*_), ..., *ϕ*(*x*_*i*_), ..., *ϕ*(*x*_*i*+*hw*_) are concatenated into a vector *z *∈ **R**^*d *× *n *^which is itself given as input to a second multi-layer perceptron. This second MLP, called *ψ *in Figure 7, has as output layer a *softmax *filtering function which allows us to consider the outputs of the neural network as probabilities. A threshold on *P*(*y*_*i *_= *loc*|*x*_*i*-*hw*_, ..., *x*_*i*_, ..., *x*_*i*+*hw*_) allows us to decide if the input is a location or not. This threshold and the hyper-parameters of the neural network are tuned on a separate validation set. Tuning this threshold away from 0.5 compensates for the fact that some *none *labels are in fact locations unknown to the HealthMap gazetteer.

## Authors' contributions

MK conceived the design and implementation of the approach, carried out its evaluation and drafted the manuscript. CF participated in the design of the approach, the collection of the data and helped draft the manuscript. JB participated in the design of the approach, was involved in the outline of its evaluation and helped draft the manuscript. All authors read and approved the final manuscript.
